# *Lactobacillus paragasseri* LG‐1 Alleviates Urticaria‐Like Symptoms in Mice via Modulation of Gut Microbiota, Hypoxanthine and Uric Acid

**DOI:** 10.1111/1751-7915.70316

**Published:** 2026-02-17

**Authors:** Qiong Wang, Zhiming Hu, Yuqi Wang, ShuPing Guo, Xinglian Zhang, Yunqing Ren, Jinjun Li, Xiaoqiong Li, Hongzhou Cui

**Affiliations:** ^1^ First Clinical Medical College of Shanxi Medical University Taiyuan China; ^2^ State Key Laboratory for Quality and Safety of Agro‐Products & Institute of Food Sciences Zhejiang Academy of Agricultural Sciences Hangzhou China; ^3^ Department of Dermatology, Children's Hospital of Shanxi Province Taiyuan China; ^4^ Department of Dermatology, Children's Hospital, Zhejiang University School of Medicine, National Clinical Research Center for Child Health Zhejiang Hangzhou China; ^5^ Zhejiang Key Laboratory of Intelligent Food Logistic and Processing Zhejiang Academy of Agricultural Sciences Hangzhou China

**Keywords:** chronic spontaneous urticaria, gut microbiota, *Lactobacillus paragasseri*, oxidative stress, purine metabolism

## Abstract

Chronic Spontaneous Urticaria (CSU) is an immunoinflammatory disorder with complex pathogenesis. Emerging evidence implicates that gut microbiota dysbiosis plays a pivotal role in this pathological network. Integrated 16S rRNA sequencing and untargeted metabolomics revealed distinct CSU‐associated signatures, including significant reductions in *Lactobacillus* abundance and elevated serum uric acid (UA) and hypoxanthine levels. Functional screening identified *Lactobacillus paragasseri* LG‐1 from breast milk as a potent purine‐metabolising strain, demonstrating significant hypoxanthine and UA degradation in vitro. In an ovalbumin (OVA)‐induced urticaria murine model, LG‐1 administration demonstrated marked reductions in serum UA and hypoxanthine concentrations, alleviated clinical manifestations, and suppressed inflammation via TLR4‐NF‐κB pathway inhibition. Moreover, it modulated gut microbial composition by promoting *Lactobacillus* proliferation while restraining pathogenic bacteria. These findings collectively established that LG‐1 exerted dual therapeutic effects through uric acid/hypoxanthine degradation and microbiome remodelling. Our study provides compelling evidence for microbiome‐targeted strategies in CSU management, highlighting LG‐1 as a promising therapeutic candidate.

## Graphical abstract

Chronic Spontaneous Urticaria (CSU) is linked to gut dysbiosis and elevated serum uric acid/hypoxanthine. *Lactobacillus paragasseri* LG‐1 alleviates urticaria by degrading these metabolites, inhibiting TLR4‐NF‐κB inflammation, and remodelling gut microbiota.

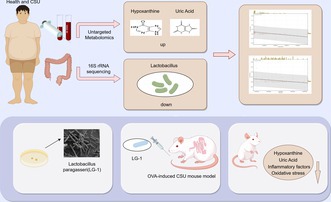

## 1 Introduction

Chronic spontaneous urticaria (CSU) is a debilitating dermatological condition characterised by recurrent episodes of intense itching, wheals, angioedema, or a combination of these symptoms (Kaplan et al. 2023). The prevalence of CSU exhibits significant geographical variation, ranging from 0.1% in North America to 1.4% in Asia, with a particularly high incidence reported in China at 2.6% (Fricke et al. 2020; Zhang et al. 2022). This condition significantly impairs patients' quality of life, often leading to sleep disturbances and, in severe cases, depressive tendencies due to persistent pain and pruritus (Gonçalo et al. 2021; Greiner et al. 2022). Although second‐generation antihistamines are first‐line for CSU, nearly 50% of patients show no symptom improvement even at standard or quadruple doses (van den Elzen et al. 2017), Beyond its impact on individuals, CSU imposes a substantial burden on families and the broader healthcare system (Licari et al. 2021). Research on the pathogenesis of CSU and the development of novel therapeutic strategies represents the current focus of investigation.

Notably, CSU is increasingly recognised as falling within the spectrum of “dysbiosis‐related diseases,” where host microbiota imbalance is linked to disease pathogenesis, as per a recent consensus (Zhang et al. 2026). Emerging evidence indicates the gut microbiota's pivotal role in CSU pathogenesis (Krišto et al. 2023). A reduction in certain bacterial taxa (e.g., *Lactobacillus and Bifidobacterium*) has been observed, while *Escherichia and Klebsiella* increased (Rezazadeh et al. 2018; Zhu et al. 2024). Probiotics (*Lactobacillus*/*Bifidobacterium*) combined with antihistamines improve symptom alleviation efficacy versus antihistamines alone in trials (Nettis et al. 2016; Bi et al. 2021). *Lactobacillus paragasseri* BBM171 attenuated OVA‐sensitised murine allergic reactions by restoring Th1/Th2 balance (Cheng et al. 2022). These findings highlight probiotic modulation of gut microbiota as a CSU therapeutic strategy.

Among the potential mechanisms linking dysbiosis to CSU, dysregulated purine metabolism also contributes to allergic asthma (Yu et al. 2016) and chronic urticaria (Kocak et al. 2022). Uric acid (UA), the end purine catabolite, forms alongside reactive oxygen species (ROS) during hypoxanthine‐to‐UA conversion via xanthine oxidase (Bortolotti et al. 2021). UA and ROS activate the NLRP3 inflammasome, promoting IL‐1β maturation and release (Bonnekoh et al. 2018; Zhou et al. 2011). UA further activates mast cells, triggering histamine and leukotriene release that exacerbates urticaria (Du et al. 2024; Spiga et al. 2017) and induces endothelial dysfunction, increasing vascular permeability, edema, and wheals (Cai et al. 2017). However, the link between aberrant purine metabolism and gut microbiota dysbiosis in CSU remains unclear.

Therefore, we analysed the gut microbiome in CSU patients versus controls via 16S rRNA sequencing and metabolomics, revealing significant downregulation of *Lactobacillus* in CSU, accompanied by elevated serum hypoxanthine and UA levels, which correlated positively. Building on this, we selected *Lactobacillus* strain LG‐1 that degraded both UA and hypoxanthine. We validated its therapeutic efficacy and mechanism in OVA‐sensitised urticaria mice.

## 2 Materials and Methods

### 2.1 Study Approval

This study was conducted in accordance with the ethical principles outlined in the Declaration of Helsinki and received formal approval from the Ethics Committee of The First Hospital of Shanxi Medical University (Approval No. KYLL‐2023‐120). Before participation, written informed consent was obtained from all human subjects. Animal experiments were performed under the approval of the Experimental Animal Welfare Ethics Committee of Zhejiang Academy of Agricultural Sciences (Approval No. 2024ZAASLA0100) and adhered strictly to the principles of the 4Rs (Replacement, Reduction, Refinement, and Responsibility) (Kang et al. 2022).

### 2.2 Study Subjects and Sample Collection

From March to May 2023, we recruited 35 patients diagnosed with CSU and 21 healthy controls (HCs), aged 18–65 years. The diagnosis of CSU was confirmed in accordance with the EAACI/GA^2^LEN/EuroGuiDerm/APAAACI guidelines (Zuberbier et al. 2022). Inclusion criteria: Patients diagnosed with CSU as per the specified criteria; no other allergic conditions, gastrointestinal diseases, autoimmune diseases, or known metabolic disorders; no use of antibiotics, probiotics, immunosuppressants, biologics, or other medications or supplements in the past month; and no systemic use of corticosteroids, antihistamines, or other urticaria‐symptom‐controlling medications in the past month. Exclusion criteria: Pregnant or lactating patients; patients with other urticaria types, such as physical, cholinergic, or stress‐induced urticaria, etc. Subjects were required to fast on the sample collection day. Faecal samples were collected for 16S rRNA gene sequencing, and serum samples were taken for untargeted metabolomics. Detailed collection and detection methods are provided in Table S1.

#### 2.2.1 The 16S rRNA Amplicon Sequencing of Human Faecal Samples and Data Analysis

Total genomic DNA was extracted from samples using hexadecyl trimethyl ammonium bromide (CTAB). Next, the V3–V4 hypervariable region of bacterial 16S rRNA was amplified using universal primers, 515F (5′‐GTGCCAGCMGCCGCGGTAA‐3′) and 806R (5′‐GGACTACHVGGTWTCT‐AAT‐3′). Amplicons were purified and sequenced on a NovaSeq platform (Illumina Co. Ltd., USA), and 250 bp paired‐end reads were generated. Raw sequences were filtered and analysed using QIIME (v.1.9.1). Then, effective sequences were used for operational taxonomic unit (OTU) clustering and species annotation using Uparse software (v.7.0.1001). To evaluate β‐diversity between the CSU and HC groups, Bray‐Curtis distance was calculated in QIIME (v.1.9.1), followed by principal coordinate analysis (PCoA). Linear discriminant analysis effect size (LEfSe) was applied to identify statistically significant biomarkers differentiating patients with CSU from HCs. Additionally, Spearman's correlation analysis between differential species and patient clinical characteristics was analysed using R package psych (v.2.1.9).

#### 2.2.2 Untargeted Metabolomics on Human Plasma Samples

Plasma samples were prepared as previously described (Wang et al. 2020). Briefly, we added 400 μL of 80% methanol aqueous solution to 100 μL plasma to precipitate proteins, followed by 5 min at room temperature, and then centrifugation at 3000 rpm for 20 min. Supernatants were collected, diluted to a 53% methanol concentration, centrifuged again for 20 min, and filtered for liquid chromatography–tandem mass spectrometry (LC–MS/MS) metabolomic assays.

LC–MS/MS analyses were performed with a Vanquish ultra‐high‐performance liquid chromatography (UHPLC) system (Thermo Fisher, Philadelphia, PA, USA) connected to an Orbitrap Q Exactive HF‐X mass spectrometer (Thermo Fisher). The samples were injected onto a Hyperil Gold column (100 × 2.1 mm, 1.9 μm) and separated using a 16‐min linear gradient at a flow rate of 0.2 mL/min (specific chromatographic and mass spectrometry conditions can be found in the Supporting Information). The raw data generated by the UHPLC–MS/MS were processed using Compound Discoverer 3.1 (Thermo Fisher) to perform peak alignment, peak picking, and metabolite quantification. Further details on the methodology can be found in the referenced section (Wang et al. 2020).

#### 2.2.3 Isolation and Screening of Purine‐Degrading *Lactobacillus* Strains

Twenty *Lactobacillus* strains with purine‐degrading potential were selected from our lab. To assess their degradation capability of UA and hypoxanthine, those strains were inoculated onto MRS media and cultured anaerobically for 48 h at 37°C. After incubation, 2 mL of the culture was centrifuged at 4000 rpm for 10 min, and the cells were washed three times with 1 mL of phosphate‐buffered saline. The washed cells were then resuspended in 750 μL of respective UA and hypoxanthine solutions and incubated at 37°C for 60 min. Following incubation, the solutions were centrifuged at 4000 rpm for 10 min to collect the supernatants. To prevent further degradation, 100 μL of 5% trifluoroacetic acid was added and mixed thoroughly. A 20 μL filtered aliquot was then taken and analysed by HPLC (Agilent Technologies, Santa Clara, CA, USA).

#### 2.2.4 Isolation and Identification of *L. paragasseri* LG‐1

DNA from *L. paragasseri* LG‐1 was extracted using a bacterial DNA extraction kit (QIAGEN China [Shanghai] Co. Ltd., Shanghai, China). After performing quality control, the DNA was randomly fragmented with an ultrasonic disruptor (Coisvar, Massachusetts, USA). Sequencing library preparation included steps such as end repair, A‐tail addition, adapter ligation, purification, and PCR amplification. Once the library was constructed, it was initially quantified and diluted using a Qubit 3.0 fluorometer (Thermo Fisher). The insert size of the library fragments was assessed using a Qsep100, and once the sizes were within the expected range, the effective concentration of the library (> 3 nM) was measured via Q‐PCR. Libraries that passed quality control were pooled into a flow cell based on their effective concentrations and the target sequencing data output. Clustering was performed using a cBOT system, followed by sequencing on a HiSeq X high‐throughput sequencing platform (Illumina Co. Ltd., USA). To assess the safety profile of the strain, the assembled genome was analysed for acquired antimicrobial resistance genes using the ResFinder 4.0 database (Bortolaia et al. 2020). Concurrently, mobile genetic elements and any associated antimicrobial resistance or virulence genes were identified using Mobile Element Finder (Johansson et al. 2021). The annotated genome of LG‐1 was subjected to functional pathway analysis using the KEGG database (Kanehisa et al. 2023). Specifically, the purine metabolism pathway was reconstructed and manually curated to identify key enzymes.

### 2.3 Animals and Experimental Design

As shown in Figure S1, a total of 30 male BALB/C mice (6–8 weeks old) were purchased from Gempharmatech Co. Ltd. (Nanjing, China) and housed in a specific pathogen‐free (SPF) facility under controlled conditions: 20°C temperature, 40%–70% humidity, and a 12‐h light/dark cycle, with ad libitum access to sterilised food and water. After a 1‐week acclimatisation period, a CSU mouse model was established following Peng's method (Peng et al. 2021). Mice were randomly assigned to six groups. Except for the normal control (NC) group, all animals received intraperitoneal injections of 0.1 mL saline containing 0.1 mg OVA and 100 mg aluminium hydroxide adjuvant on days 1 and 10. From days 8 to 28, mice were orally administered purified water, LG‐1 solution (10^9^ CFU/mL, 200 μL/day), or loratadine solution (4 mg/kg) for 21 consecutive days. On day 29, faecal samples were collected, after which the mice were anaesthetised using 1.5% sodium pentobarbital. Blood samples were collected, followed by anaesthesia with 1.5% sodium pentobarbital. Blood samples were then obtained, and mice were euthanised for the collection of liver, dorsal skin, spleen, and intestinal tissues.

#### 2.3.1 DNA Extraction and 16S rRNA Amplification

After collecting mouse faeces on day 28, microbial DNA was extracted. All methods used to analyse gut microbiota diversity and species composition were previously described. PCR on microbial DNA was conducted using the 16S rRNA V3–V4 region using differential tag barcodes and 341F (5′‐CCTAYGGGRBGCASCAG‐3′) and 806R (5′‐GGACTACNNGGGGTATCTAAT‐3′) universal primers. Once amplified, PCR products were extracted from gels and purified according to QIAquick Gel Extraction kit (QIAGEN China [Shanghai] Co. Ltd., Shanghai, China) instructions. Purified DNA was then used to construct a library using the Illumina TruSeq Nano DNA Library prep kit (Illumina Co. Ltd., USA). High‐throughput sequencing was performed on the Illumina Novaseq S6000 platform (Illumina Co. Ltd.). Sequences were then spliced, quality controlled, and filtered, and chimeras removed using QIIME software to generate valid data.

#### 2.3.2 Short Chain Fatty Acid (SCFA) Analysis and Purine‐Degradation by HPLC

SCFA content in faeces was detected by gas chromatography (GC) (Fuli, Shanghai, China) equipped with a capillary DB‐FFAP (30 m × 0.25 mm × 0.25 μm) column (Agilent, Bollinger, Germany). Trans‐2‐butenoic acid was used as an internal standard (Liu, Li, et al. 2020). The serum (50 μL) was combined with 200 μL of 0.08 mol/L sulfuric acid solution and 100 μL of 10% sodium tungstate solution. The mixture was vortexed and subjected to ultrasonic treatment in an ice‐water bath for 2 min. Subsequently, the sample was centrifuged at 12,000 rpm for 10 min at 4°C. The concentrations of uric acid (UA), hypoxanthine, and xanthine in the serum and faeces were determined using high‐performance liquid chromatography (HPLC) with a CAPCELL PAK ADME S5 column (4.6 mm i.d. × 250 mm, Shiseido, Tokyo, Japan). The mobile phase consisted of a gradient of 50 mM ammonium acetate solution (pH = 4.65), with a flow rate of 1 mL/min and detection at 254 nm. The total elution time was 20 min.

#### 2.3.3 Skin Oxidative Stress Indicators and Xanthine Oxidase (XOD) Activity

Dorsal tissues were homogenised in cold normal saline (10%, w/v), centrifuged at 3000 rpm for 15 min at 4°C, and supernatants retained. Oxidative stress indices (superoxide dismutase [SOD], catalase [CAT], and malondialdehyde [MDA]) were determined in skin tissue using kit instructions (Beijing Boxbio Science & Technology Co. Ltd., Beijing, China). Serum samples were processed for XOD activity following kit guidelines (Beijing Boxbio Science & Technology Co. Ltd.). Extracted colon tissue was weighed, with subsequent steps performed using kit instructions. Specifically, tissue blocks were added to a pre‐cooled extraction solution at a 1:10 ratio, mechanically homogenised, centrifuged, and supernatants collected for XOD activity assays. Protein concentrations for calibration were determined using the bicinchoninic acid method.

#### 2.3.4 Disease Phenotyping and Pathological Analysis

##### 2.3.4.1 Scratching Behaviour Assessment

The severity of itching (including scratching latency, duration, and frequency) was monitored within 30 min following the second immunisation in all groups. Signs of itching included head scratching with the front paws, scratching of the trunk post‐immunisation, and biting of various body parts (Choi et al. 2018).

##### 2.3.4.2 Histopathological Examination

Dorsal skin samples were fixed in 10% paraformaldehyde for 48 h, dehydrated using ethanol, and embedded in paraffin wax. Tissue sections of 3 μm thickness were prepared and treated with xylene and ethanol, followed by staining with haematoxylin and eosin (H&E) (Wuhan Servicebio Technology Co. Ltd., Wuhan, China) to evaluate edema and inflammatory cell infiltration. Images were randomly captured by a pathologist using light microscopy (Nikon Eclipse E100, Tokyo, Japan) at ×100 magnification (×10 ocular and ×10 objective lenses). The degree of edema, telangiectasia, and inflammatory cell infiltration was assessed using a four‐point grading system (Peng et al. 2021).

##### 2.3.4.3 Mast Cell Degranulation Analysis

Skin tissues were stained with toluidine blue solution (Wuhan Servicebio Technology Co. Ltd.) at 37°C for 10 min and sealed in resin. Stained MCs were enumerated under microscopy (Nikon Eclipse E100) by randomly observing three fields/section at ×100 magnification.

#### 2.3.5 Immune Molecular Profiling

##### 2.3.5.1 Serum Immunoglobulin E (IgE) Detection

The collected blood samples were centrifuged at 4000 rpm for 10 min to separate the serum. Serum immunoglobulin E (IgE) levels were quantified using ELISA kits (JianChen Bioengineering Institute, Nanjing, China). The optical densities were measured at 450 nm using a microplate reader (Thermo Fisher, Waltham, MA, USA), and IgE concentrations were calculated based on the standard curve.

##### 2.3.5.2 Reverse Transcription‐Quantitative Polymerase Chain Reaction (RT‐qPCR)

The mRNA expression levels of TNF‐α, IL‐1β, IL‐10, NF‐κB, IL‐4, and TLR4 in skin tissues were quantified using qRT‐PCR. cDNA was synthesised using the PrimeScript RT reagent kit (Beijing Boxbio Science & Technology Co. Ltd., Beijing, China), and the resulting cDNA was diluted 10‐fold to serve as templates in the qRT‐PCR. The reactions were carried out using a real‐time fluorescent quantitative PCR instrument (Bio‐Rad Laboratories, CA, USA). The qPCR protocol included an initial incubation at 50°C for 2 min, followed by denaturation at 95°C for 10 min, and 40 amplification cycles at 95°C for 15 s and 60°C for 30 s. A final cycle included 95°C for 15 s and 60°C for 1 min. Glyceraldehyde 3‐phosphate dehydrogenase (Beijing Tsingke Biotech Co. Ltd., Beijing, China) was used as the internal control. The relative mRNA expression levels were calculated using the $2^{-∆∆C_{t}}$ method. The primers used are listed in Table S2.

##### 2.3.5.3 Immunohistochemical Analysis of Inflammatory Pathways

Sections were deparaffinised with xylene, followed by a graded ethanol series (100%, 95%, 85%, and 75%) for 5 min each, and then rinsed in distilled water for 5 min. Antigen retrieval was carried out by heating the sections in citrate buffer (pH 6.0) at 95°C for 20 min. Endogenous peroxidase activity was blocked with 3% hydrogen peroxide for 10 min. The sections were then incubated overnight at 4°C with primary antibodies against TLR4 (1:200) and NF‐κB (1:100). After washing, the sections were incubated with biotinylated secondary antibodies followed by streptavidin‐HRP conjugate. Signal detection was performed using DAB (Diaminobenzidine) substrate, and the sections were counterstained with haematoxylin. Positive staining was observed under light microscopy at ×200 magnification. The staining intensity was evaluated semi‐quantitatively by calculating the percentage of positively stained cells in randomly selected fields.

### 2.4 Statistical Analysis

All data were analysed using GraphPad Prism 8.0. Student's *t*‐test was used for comparisons between two independent groups, while one‐way or two‐way analysis of variance (ANOVA) followed by Tukey's multiple comparison test was applied to evaluate differences among three or more groups. Data are presented as the mean ± SEM. Statistical significance was set at *p < 0.05, **p < 0.01, ***p < 0.001 and ****p < 0.0001 for pairwise comparisons.

## 3 Results

### 3.1 Gut Microbiota Dysbiosis in Patients With CSU

**FIGURE 1:**
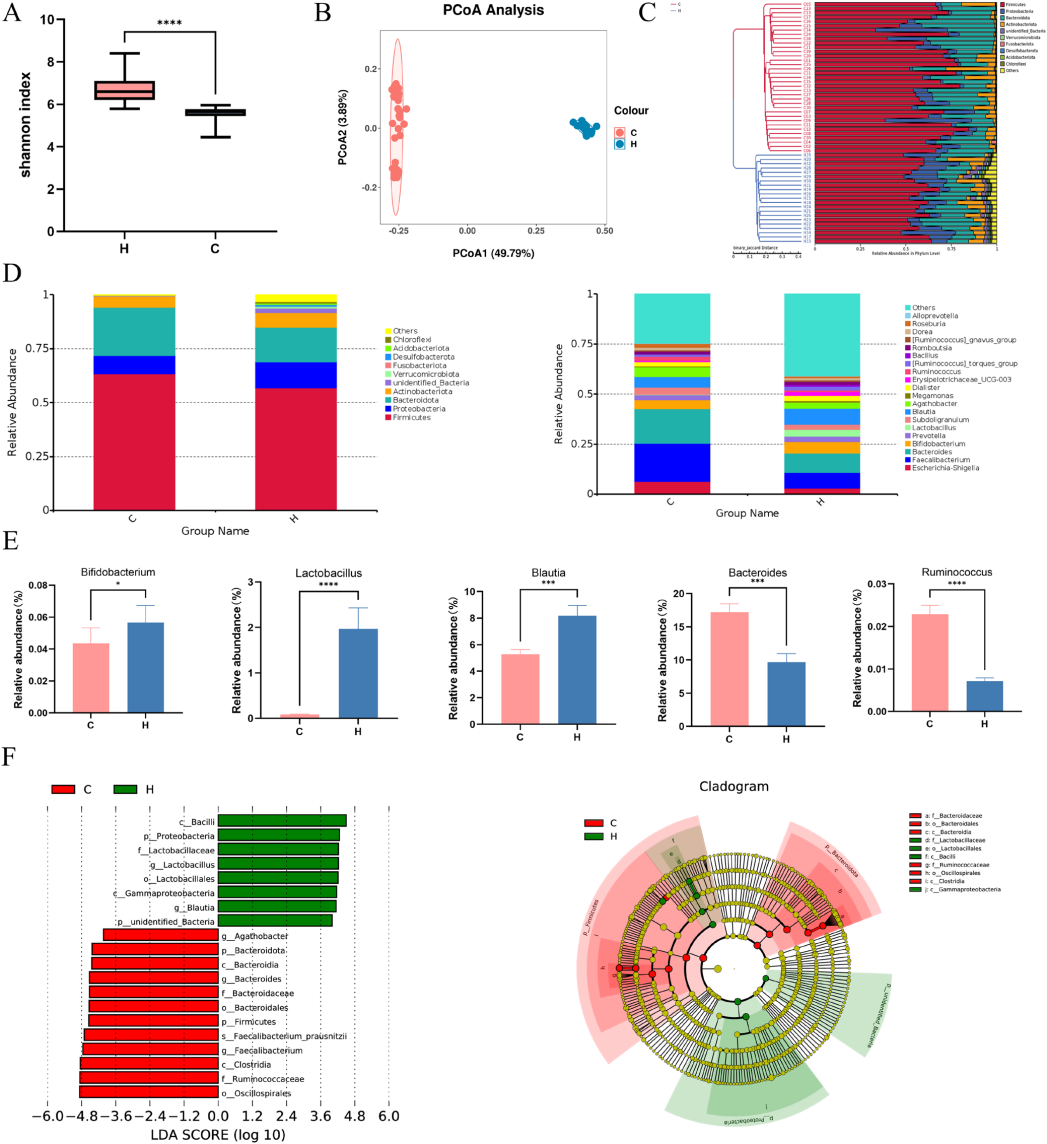
The difference in gut microbiota between patients with CSU (C) (*n* = 35) and HCs (H) (*n* = 21). (A) α‐diversity (Shannon diversity) of gut microbiota. (B) Principal Co‐ordinates analysis (PCoA) on gut microbiota structures. (C) UPGMA clustering tree. (D) The composition of gut microbiota at the genus level. (E) LEfSe analysis showing relative bacterial taxa abundance values between groups. (F) Five bacterial genera had significant differences at the genus level between patients with CSU and HCs. Data were expressed as the mean ± SEM. *p < 0.05, ***p < 0.001, ****p < 0.0001.

Faecal 16S rRNA sequencing revealed distinct gut microbial profiles between 35 CSU patients and 21 HCs. α‐diversity indicated reduced microbial richness and evenness in CSU patients (*p* < 0.05), except for increased Goods coverage (Figures 1A, S2A). β‐diversity via PCoA showed group separation, supported by phylum‐level UPGMA clustering (Figures 1B,C, S2B). At phylum level, Proteobacteria were depleted in CSU vs. HCs (*p* < 0.05), though Firmicutes and Proteobacteria maintained > 50% abundance (Figure 1D). Genus‐level quantification identified reductions in *Lactobacillus*, *Blautia*, and *Bifidobacterium* (*p* < 0.05), with elevated *Ruminococcus* and *Bacteroides* in CSU (Figure 1E). Linear discriminant analysis (LEfSe) found 20 enriched genera (LDA > 4), Oscillospirales and Ruminococcaceae defined CSU, versus Bacilli, Proteobacteria, and Lactobacillaceae in HCs (Figure 1F).

### 3.2 Serum Hypoxanthine and Uric Acid Dysregulation in CSU

**FIGURE 2:**
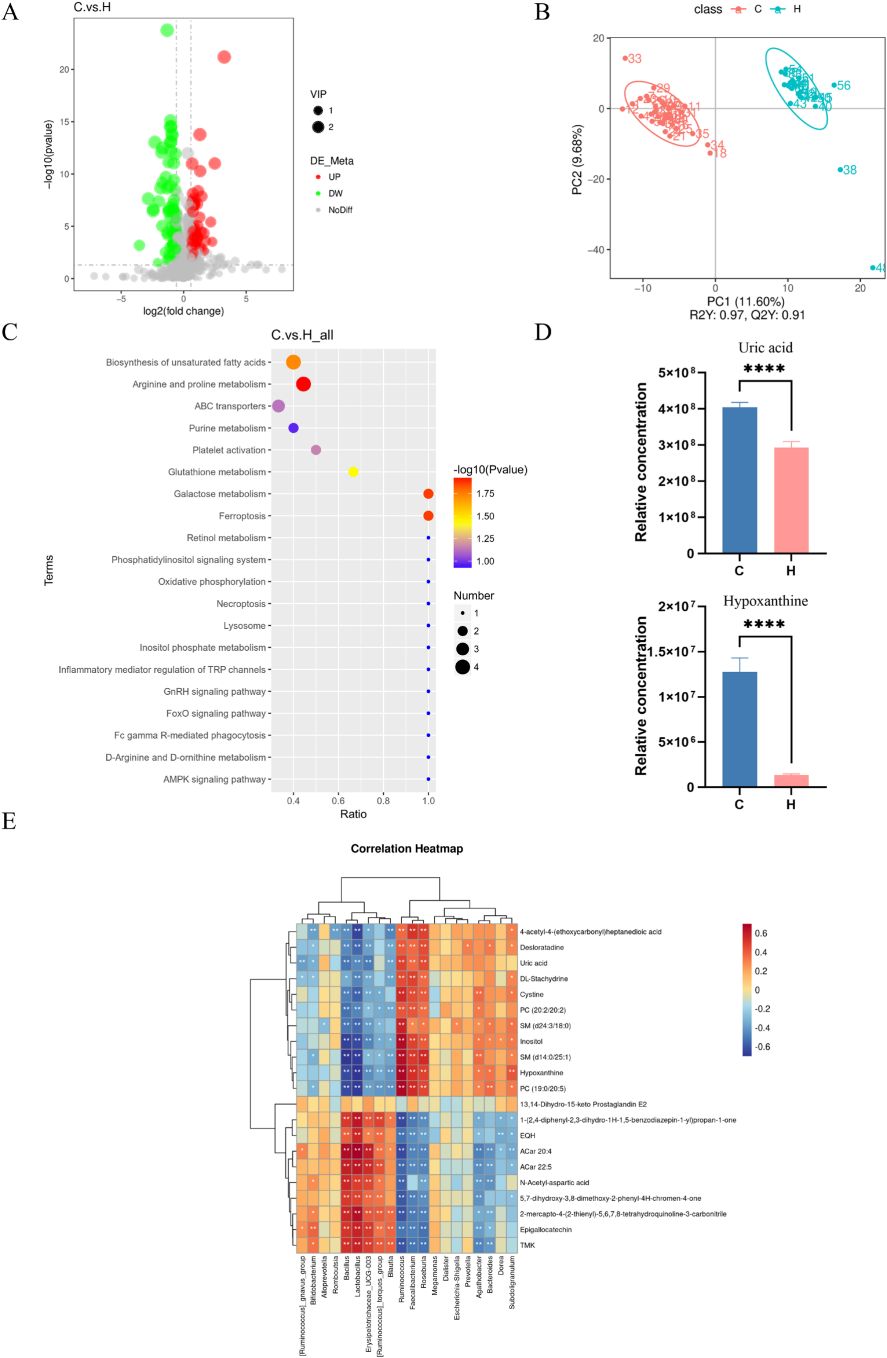
Characteristics of metabolomics in serum. (A) Volcano plot showing identified metabolites in different ion modes. (B) OPLS‐DA score plot showing CSU and HC groups. (C) KEGG enrichment analysis of changed metabolites. (D) Relative uric acid and hypoxanthine serum concentrations in CSU and HC groups. (E) Heat map showing differential gut microbiota correlations between serum metabolites and genus levels. Only significant correlations (*p* ≤ 0.05) are coloured. Positive correlations are indicated in red and negative correlations in blue. ****p < 0.0001. Data were expressed as the mean ± SEM.

Emerging evidence indicates critical host‐microbiota metabolic crosstalk in immune‐mediated dermatoses including CSU (Zhu et al. 2024). Untargeted serum metabolomics of 35 CSU patients versus 21 HCs identified 150 significantly altered compounds (*p* < 0.05; Figure 2A). Key altered classes included lipids, organic acids, unsaturated fatty acids, and nucleotides. Analysis revealed 69 downregulated metabolites (FC < 1; e.g., phosphatidylcholine, ornithine, hydroxyproline, epicatechin) and 81 upregulated species (FC > 1; e.g., xanthine, arachidonic acid, cholesterol sulfate, cysteine). Hypoxanthine showed the highest variable importance in projection (VIP) score, indicating its pivotal role in CSU pathogenesis.

Orthogonal partial least squares‐discriminant analysis (OPLS‐DA) confirmed robust group separation (*R*2Y = 0.97, *Q*2 = 0.91), underscoring robust separation of serum metabolic profiles (Figure 2B). Pathway enrichment analysis identified eight significantly dysregulated metabolic pathways (*p* < 0.05), including purine metabolism, unsaturated fatty acid biosynthesis, arginine/proline metabolism, ABC transporters, platelet activation, glutathione metabolism, galactose metabolism, and ferroptosis (Figure 2C). Of these, purine metabolism emerged as a key hub, with uric acid (UA) and hypoxanthine serving as central nodes whose dysregulation may drive metabolic imbalances (Figure 2D).

Spearman's correlation analysis revealed significant associations between gut microbiota and serum metabolites (Figure 2E). Interestingly, *Lactobacillus* and *Blautia* displayed strong negative correlations with hypoxanthine and UA levels, whereas *Ruminococcus* exhibited significant positive associations. Notably, Bifidobacterium abundance was positively correlated with epicatechin, suggesting microbiota‐mediated regulation of antioxidant pathways.

### 3.3 LG‐1 Demonstrates Hypoxanthine and Uric Acid Degradation

**FIGURE 3:**
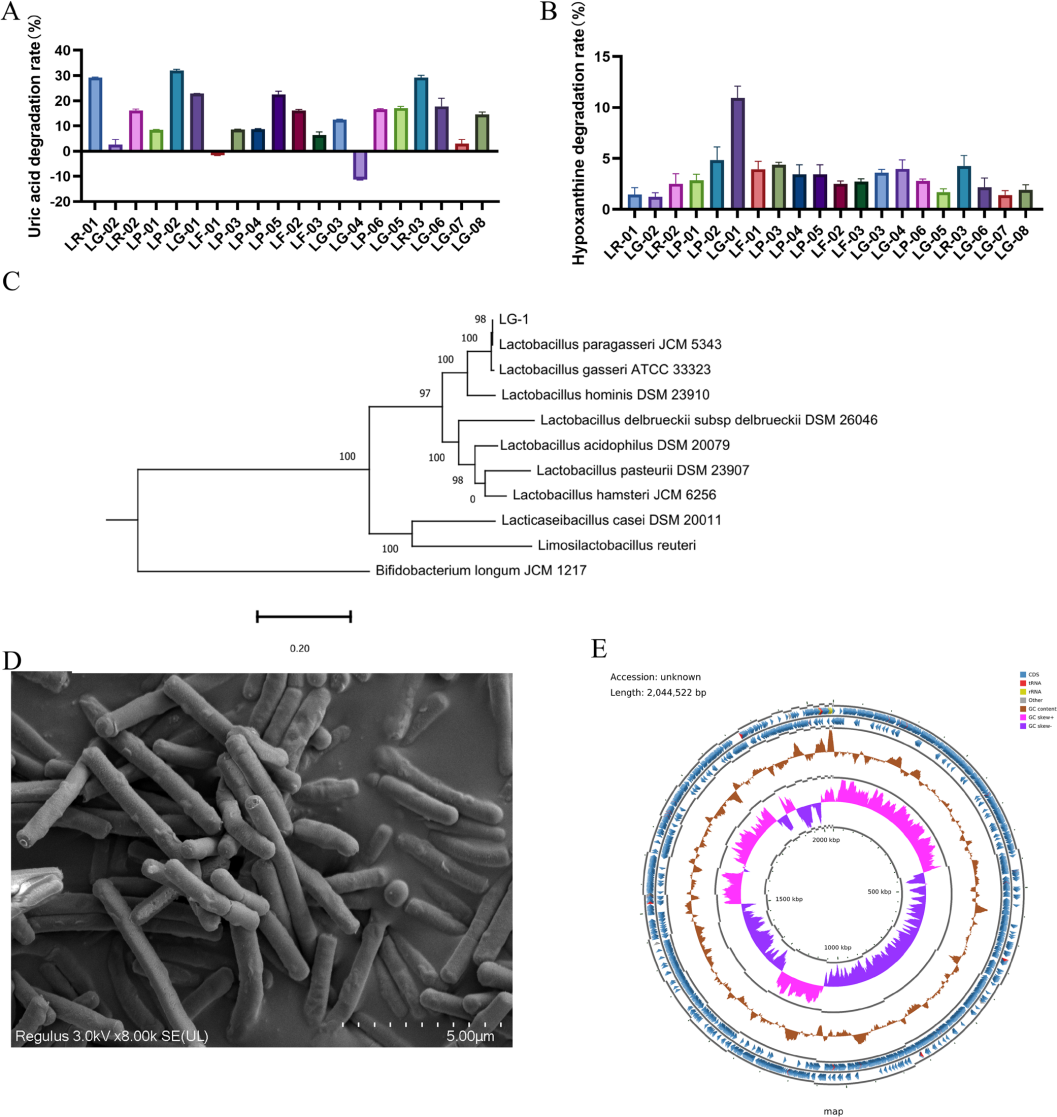
Screening and identification of LG‐1 strain. (A) The effects of 20 *Lactobacillus* strains on uric acid and hypoxanthine degradation. (B) The genetic phylogenetic tree. (C) Scanning electron microscope image showing LG‐1 morphology. (D) Circular map of *Lactobacillus paragasseri* LG‐1.

We identified 20 candidate strains from our laboratory collection capable of degrading uric acid (UA) and hypoxanthine, with strain LG‐1 (*Lactobacillus paragasseri*, isolated from breast milk) exhibiting exceptional activity (22.8% UA degradation and 10.94% hypoxanthine degradation) (Figure 3A). Comprehensive phylogenetic characterisation revealed LG‐1's taxonomic position within the Lactobacillus genus. Whole‐genome sequencing alignment demonstrated 99.4% identity with *Lactobacillus paragasseri* type strains, while scanning electron microscopy (SEM) confirmed typical morphological characteristics including rod‐shaped cells with smooth surfaces and rounded ends (Figure 3C). The phylogenetic analysis based on 16S rRNA gene sequences further validated its classification within the *L. paragasseri* clade (Figure 3B). Genomic analysis revealed a single circular chromosome of 2,044,522 bp with 36.74% GC content (Figure 3D), containing 2007 protein‐coding genes and 60 RNA genes (55 tRNA, 5 rRNA). In‐depth genomic safety assessment confirmed the absence of known virulence factors and acquired antibiotic resistance genes. Furthermore, analysis of its genomic architecture identified common mobile genetic elements such as insertion sequences and prophage regions; critically, no toxin genes were detected within these regions. Beyond establishing this safety profile, we sought to genomically define the functional capacity of LG‐1, particularly its potential to interact with host purine metabolism. To this end, a detailed KEGG pathway analysis was performed, which revealed a purine metabolism pathway in strain LG‐1. This includes genes encoding critical enzymes for both degradation and biosynthesis, such as adenine deaminase (LG‐1_g_00010, K01486) for purine degradation; hypoxanthine phosphoribosyltransferase (LG‐1_g_00465, K00760) and adenine phosphoribosyltransferase (LG‐1_g_01750, K00759) for the purine salvage pathway (Table S3). This suggests LG‐1 is adept at utilising and modulating purine compounds in its environment, positioning it as a potential modulator of host purine homeostasis.

### 3.4 LG‐1 Modulates Gut Microbiota in Urticaria Mice

**FIGURE 4:**
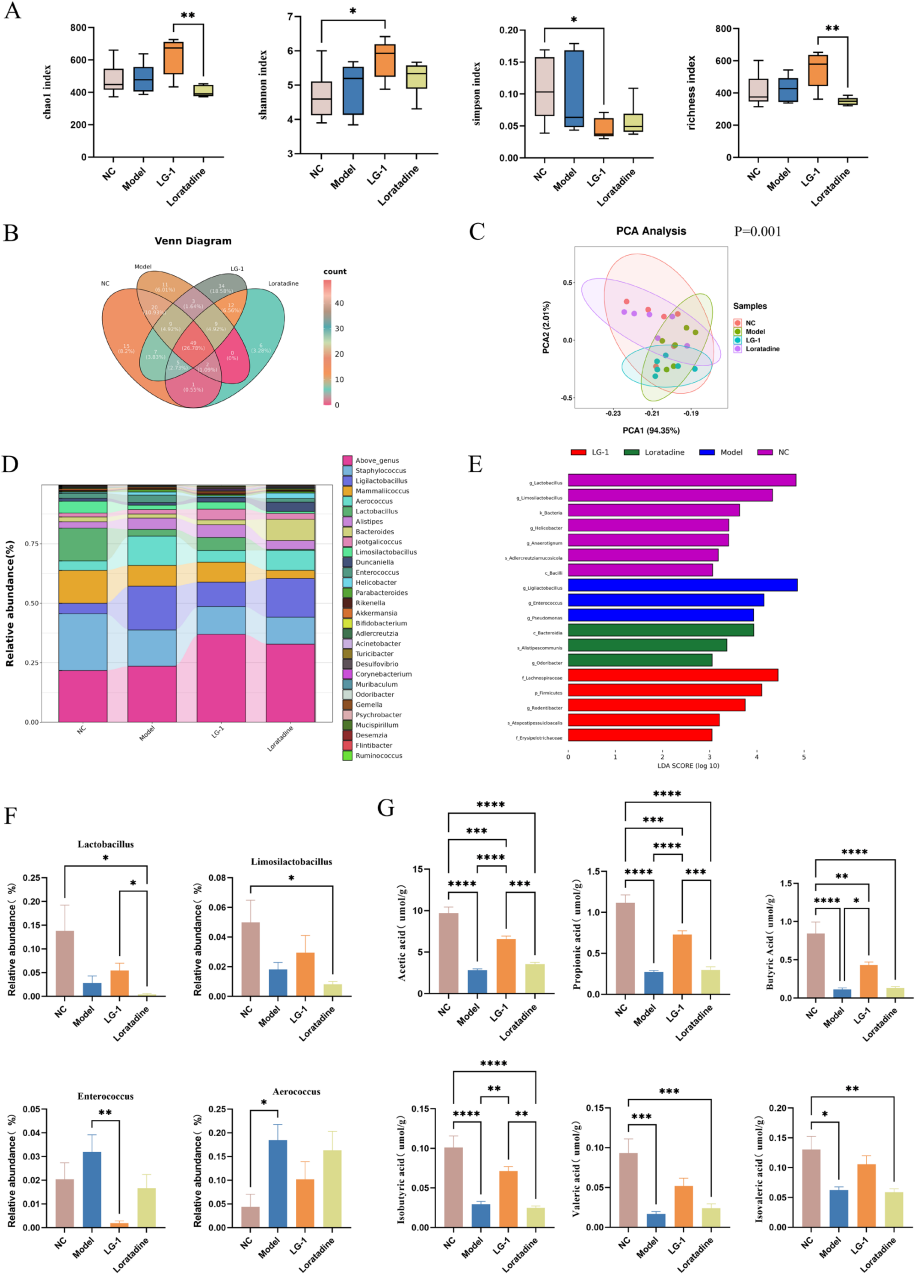
Gut microbiota structure and composition in different mouse groups. (A) α‐diversity of gut microbiota in mice. (B) Venn diagram showing OTUs. (C) Principal coordinate analysis. (D) Relative gut microbiota abundance at the phylum level. (E) LEfSe results showing significant differential biomarkers. (F) Significant analysis at the genus level. (G) Short‐chain fatty acid concentrations in mouse faeces. *p < 0.05, **p < 0.01, ***p < 0.001, ****p < 0.0001. Compared with the model group.

16S rRNA sequencing revealed that LG‐1 supplementation significantly increased gut microbiota diversity in mice with urticaria‐like symptoms. Interestingly, supplementation with the LG‐1 strain significantly increased the Chao1 and Richness indices compared to the loratadine group, with a slight increase observed relative to the model group. Additionally, the Shannon index was significantly higher in the LG‐1 group than in the NC group and also increased compared to the model group, while the Simpson index was significantly lower in the LG‐1 group than in the NC group and decreased relative to the model group (Figure 4A). These results indicate that supplementation with the LG‐1 strain enhances the α‐diversity of the gut microbiota. Venn diagram analysis identified a total of 183 operational taxonomic units (OTUs) (Figure 4B). Among these, the LG‐1 group had the highest number of unique OTUs (34), followed by the NC group (15), the model group (11), and the loratadine group (6). Principal coordinate analysis (PCoA) revealed a high degree of overlap between the LG‐1 and NC groups, further suggesting that LG‐1 exerts regulatory effects on the gut microbiota (Figure 4C).

At the genus level, LG‐1 treatment altered gut microbiota composition in mice with urticaria. LG‐1 increased relative *Lactobacillus* and *Limosilactobacillus* abundance; while conversely, relative *Enterococcus* and *Aerococcus* abundance was decreased (Figure 4D). Lachnospiraceae, Firmicutes, *Rodentibacter*, *Atopostipessuicloacalis* and Erysipelotrichaceae were the dominant genera in LG‐1 mice, *Ligilactobacillus*, *Enterococcus*, and *Pseudomonas* were dominant genera in model mice (Figure 4E). Following LG‐1 treatment, short‐chain fatty acid (SCFA)‐producing bacteria were markedly enriched and showed an upward trend in *Lactobacillus* and *Limosilactobacillus* levels (Figure 4F). This indicated that SCFA‐producing bacteria played important roles in urticaria treatment with LG‐1. In consideration of this, GC detected SCFAs in mice faeces after treatment. These results revealed that acetic acid, propionic acid, butyric acid, isobutyric acid, isovaleric acid, and valeric acid levels in model group faeces were significantly lower than in the control group. Compared to the model group, LG‐1 significantly increased acetic acid, propionic acid, and isobutyric acid concentrations, which were superior to the positive control drug Loratadine (Figure 4G).

### 3.5 LG‐1 Normalises Purine Metabolism in Urticaria Mice

**FIGURE 5:**
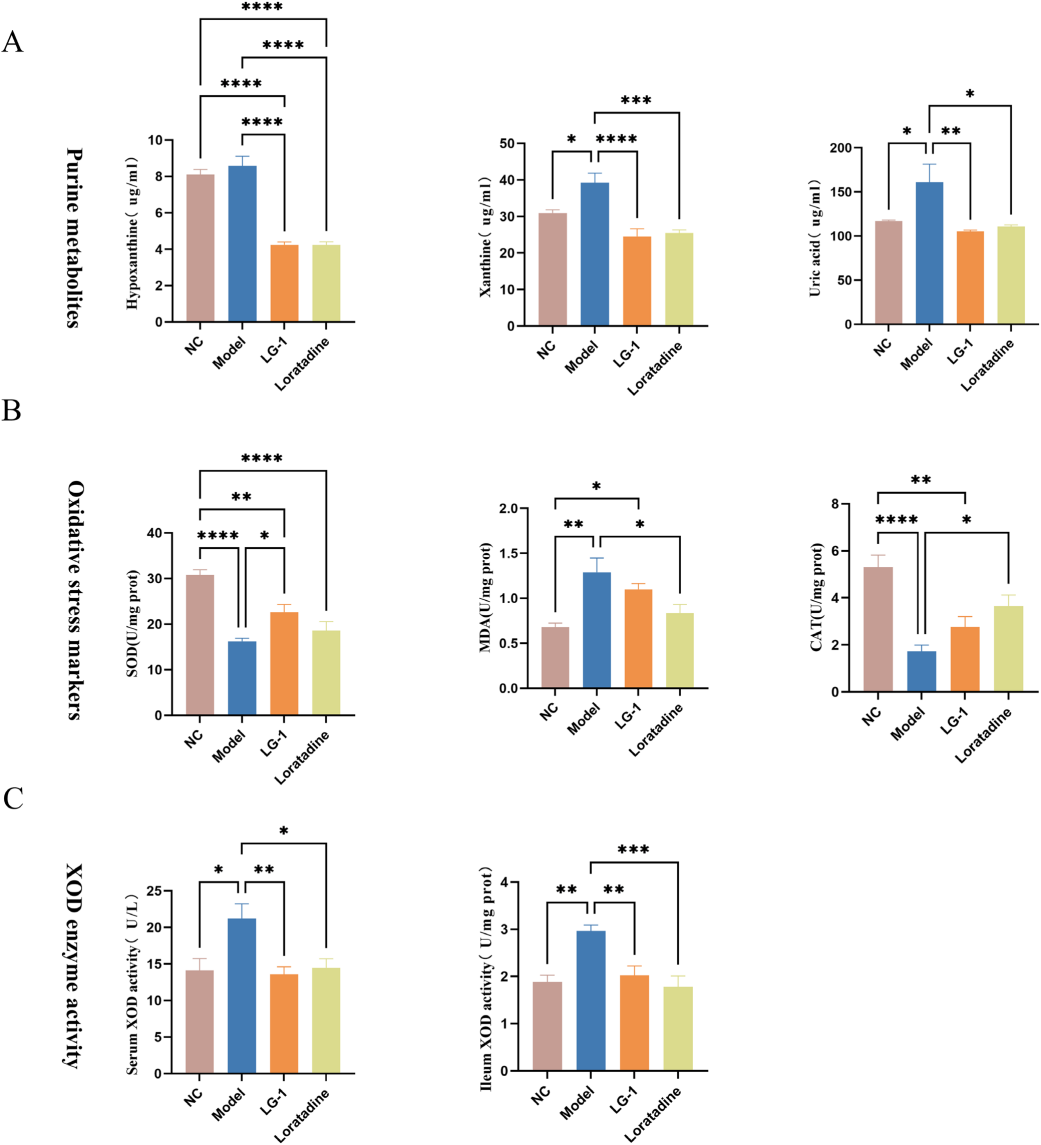
Changes of purine metabolism, oxidative stress, and inflammatory factors induced by LG‐1 in mice. (A) Changes in purine metabolites in serum. (B) Oxidative stress markers in dorsal skin samples from different mouse groups. (C) XOD levels in ileum and serum samples from different groups. Data were expressed as the mean ± SEM. *p < 0.05, **p < 0.01, ***p < 0.001, ****p < 0.0001.

Then, we detected purine metabolites in mouse serum and faeces. As shown in Figure 5A, We analysed UA serum levels and found that urticaria markedly increased UA and xanthine, while LG‐1 or loratadine abrogated elevated UA concentrations. When compared with the NC group, no differences in hypoxanthine levels were observed in mice with urticaria. In faeces, hypoxanthine levels were significantly increased in mice with urticaria, but were decreased after LG‐1 intervention (Figure S3). Thus, the purine metabolism pathway was altered in urticaria, while LG‐1 treatment appeared to normalise purine metabolism.

### 3.6 LG‐1 Attenuates Oxidative Stress in Urticaria Mice

Compared to normal mice, the model mice exhibited significantly increased MDA levels and significantly decreased SOD and CAT activities (*p* < 0.05), indicating that alterations in oxidative stress levels are involved in the pathogenesis of CSU. Following LG‐1 intervention, SOD levels were significantly increased (*p* < 0.05). Although no significant differences were observed in MDA and CAT levels compared to the model group, MDA showed a decreasing trend, while CAT exhibited an increasing trend (Figure 5B). In the positive control group treated with loratadine, both SOD and MDA levels were significantly reduced, while CAT levels were significantly elevated (*p* < 0.05). Additionally, we measured xanthine oxidase (XOD) levels in serum and ileum samples from mice with urticaria‐like symptoms (Figure 5C). Compared to the normal group, XOD levels were significantly increased, but were significantly reduced after intervention with either LG‐1 or loratadine (*p* < 0.05). These results demonstrate that LG‐1 effectively alleviates oxidative stress levels in urticaria mice.

### 3.7 LG‐1 Ameliorates CSU Pathophysiology in Mice

**FIGURE 6:**
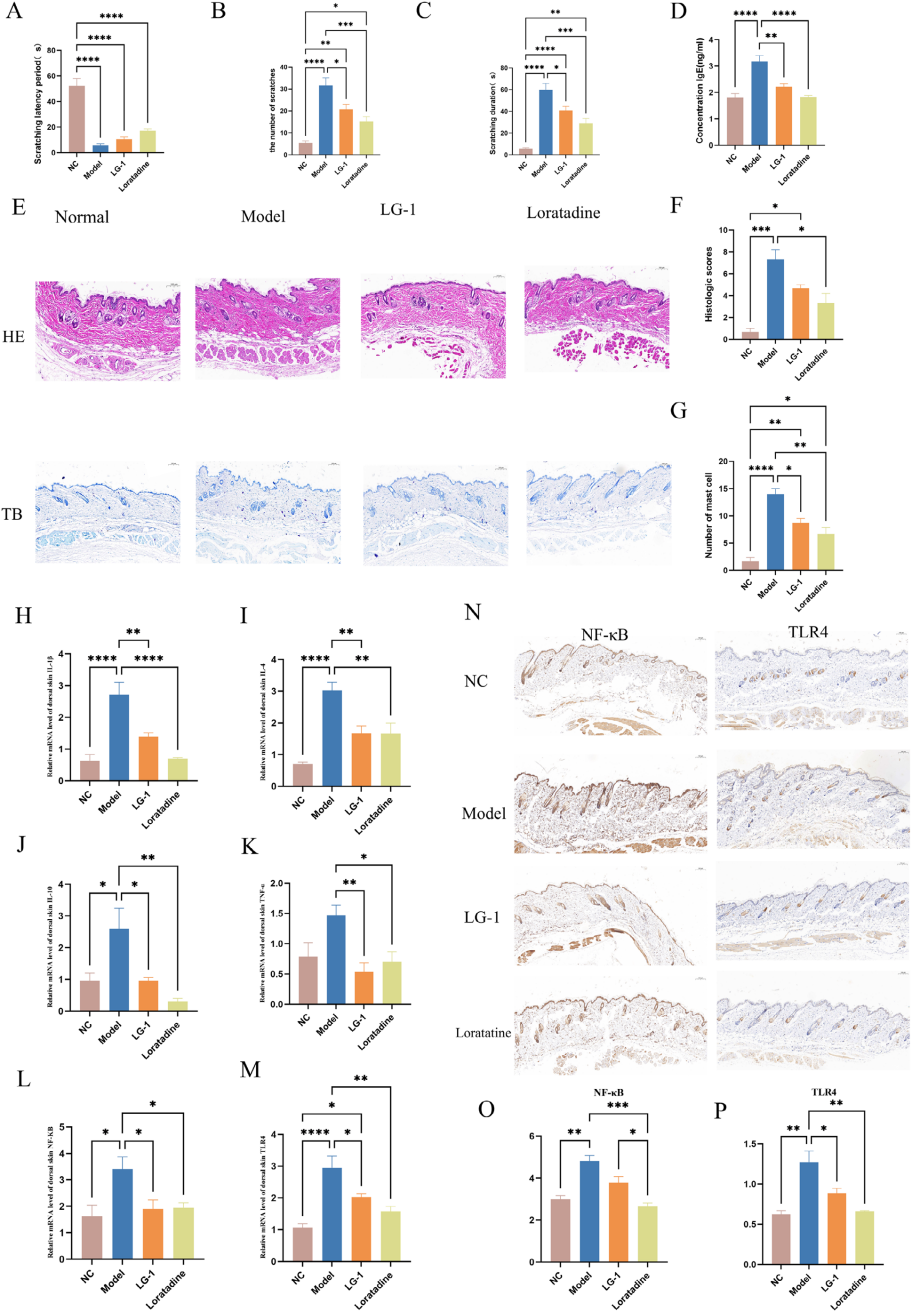
Study on OVA/aluminium hydroxide‐induced urticaria in mice. (A–C) Scratching behaviour analysis. (D) Serum IgE levels across different groups. (E) Representative images of dorsal skin samples stained with haematoxylin and eosin (HE) and toluidine blue (TB). (F) Histological scoring of urticaria severity. (G) Data on mast cell degranulation. (H‐M) The mRNA expression levels of the inflammatory cytokines (IL‐1β, IL‐4, IL‐10, and TNF‐α, NF‐κB and TLR4) in mouse skin tissue. (N–P) Skin tissue sections were stained by IHC. Representative skin tissue sections (scale = 100 μm, magnification ×200) were stained, and NF‐ĸB and TLR4 expression in skin tissue sections examined. Data were expressed as the mean ± SEM. *p < 0.05, **p < 0.01, ***p < 0.001, ****p < 0.0001. Skin tissue sections were stained by IHC. Representative skin tissue sections (scale = 100 μm, magnification ×200) were stained, and NF‐ĸB and TLR4 expression in skin tissue sections examined.

We next assessed LG‐1 effects on OVA/aluminium hydroxide‐induced urticaria in mice. In scratching tests, after stimulation, obvious scratching reactions were observed. When compared to the model group, LG‐1 significantly improved skin scratching behaviours in mice (Figure 6A–C). Decreased IgE excretion was observed in LG‐1 and loratadine treated mice when compared to model animals (Figure 6D). H&E staining indicated that the NC group mice with urticaria exhibited significant dermal edema, increased granular layer thickness, and dilated capillaries in skin tissue, along with inflammatory cell infiltration. As expected, when compared to the model group, pathological skin tissue morphology in LG‐1 and the loratadine mice was improved to varying degrees (Figure 6E), while histological scores were significantly reduced (Figure 6F). MCs are crucial effector cells in urticaria pathogenesis. Toluidine blue staining was performed to analyse MC numbers and degranulation (Figure 6G). Therefore, LG‐1 exerted certain protective effects in mice with urticaria. Compared to the NC group, the CSU model group exhibited significantly elevated mRNA levels of IL‐4, IL‐10, IL‐1β, NF‐κB, and TLR4 (*p* < 0.05), along with a slight increase in TNF‐α. However, treatment with LG‐1 and loratadine markedly reduced the expression levels of these mRNAs (Figure 6H–M). Additionally, immunohistochemical (IHC) results demonstrated a significant increase in the expression of NF‐κB and TLR4 in the skin tissues of the model group compared to the NC group (Figure 6N–P). These findings suggest that the activation of these pathways plays a crucial role in the inflammatory response associated with urticaria. In contrast, LG‐1 reduced the expression of NF‐κB and TLR4 in the skin, with a particularly significant difference observed in TLR4 levels. These results indicate that LG‐1 may exert a notable anti‐inflammatory effect on mouse skin tissue by modulating the TLR4/NF‐κB signalling pathway, thereby reducing the levels of inflammatory cytokines and alleviating the inflammatory response in the CSU mouse model.

## 4 Discussion and Conclusions

In our study, we observed significant differences in gut microbiota composition and serum biomarkers between patients with CSU and HCs. Notably, there was a marked downregulation of beneficial *Lactobacillus* species in the gut microbiota, accompanied by elevated serum levels of hypoxanthine and UA serum levels. To explore potential therapeutic interventions, we isolated and cultured *L. paragasseri* LG‐1, which demonstrated a significant capacity to reduce hypoxanthine and UA levels in vitro. Furthermore, LG‐1 administration alleviated urticaria‐like symptoms in an OVA/aluminium hydroxide‐induced mouse model, correlating with reduced hypoxanthine and UA levels, diminished inflammation, and decreased oxidative stress. Additionally, LG‐1 ameliorated gut microbiota dysbiosis, further supporting its therapeutic potential.

Gut microbiota dysbiosis is a hallmark of chronic inflammatory skin diseases including atopic dermatitis, psoriasis, acne, and CSU (Kapoor et al. 2022; Sánchez‐Pellicer et al. 2022; Lindberg and Söderquist 2017). Specifically, CSU gut microbiota has reduced diversity, diminished SCFA production, and elevated *Klebsiella pneumoniae* (Zhu et al. 2024; Wang et al. 2020). In our study, CSU patients exhibited significant alterations in gut microbiota structure, marked by decreased α‐diversity and a decline in the relative abundance of beneficial bacteria, such as *Bifidobacterium*, *Lactobacillus*, and *Blautia*. Conversely, an increased abundance of Bacteroides was observed in both CSU patients and mouse models, which contrasts with some earlier reports that found reduced levels of Bacteroides in patients with CU (Rezazadeh et al. 2018; Nabizadeh et al. 2017) or CSU (Wang et al. 2020; Liu et al. 2021). This discrepancy may stem from diet, environment, or antibiotic use. Enterococcus genus pathogens increased in urticaria mice. These align with prior CSU and allergy research (Zhu et al. 2024; Wang et al. 2020), further underscoring the strong association between gut microbiota dysbiosis and the pathogenesis of CSU.

The efficacy of LG‐1 in alleviating these abnormalities can be interpreted through a combination of direct and microbiota‐mediated effects. The reduction in serum uric acid and hypoxanthine is most parsimoniously explained by the direct degradation of these purines by LG‐1 within the intestinal lumen, a capability confirmed in vitro and supported by genomic identification of a complete purine metabolism pathway in LG‐1, encompassing key genes for purine degradation and synthesis. Hypoxanthine via xanthine to UA by the enzyme xanthine oxidoreductase (XOR). XOR serves as a critical, rate‐limiting enzyme in purine catabolism, catalysing the conversion of hypoxanthine to xanthine and subsequently xanthine to UA. This enzymatic process concurrently generates reactive oxygen species (ROS) as a byproduct, contributing to oxidative stress (Bortolotti et al. 2021). Elevated levels of UA, xanthine, and hypoxanthine have been documented in blood and urine samples from patients with CSU and allergic patients (Yu et al. 2016; Kocak et al. 2022; Tao et al. 2019), findings that align with our observations. The increased hypoxanthine concentration may result from either impaired enzymatic conversion of hypoxanthine to UA or inhibition of XOR. Additionally, hypoxanthine levels can rise in response to hypoxia, which elevates AMP, a precursor of hypoxanthine. In CSU models, elevated XOR activity and UA levels suggest that the accumulation of hypoxanthine may be driven by cellular hypoxia induced by oxidative stress. This was supported by significantly reduced levels of superoxide dismutase (SOD) and catalase (CAT), alongside markedly elevated malondialdehyde (MDA) levels in the model group. Upregulated XOR activity and its byproducts have been implicated in a range of pathological conditions, including hyperuricemia, gout, ischemia–reperfusion injury, adipogenesis, cardiovascular disease, and cancer. Its specific role in the pathogenesis of CSU remains to be fully elucidated. Of particular relevance, XOR inhibition has demonstrated therapeutic potential in ameliorating oxidative stress and inflammation. This observation suggests a potential self‐perpetuating cycle wherein CSU progression may be exacerbated through XOR‐mediated dysregulation of purine metabolism and oxidative stress pathways.

Gut microbiota critically influences UA levels via metabolites like SCFAs and LPS, driving hyperuricemia pathogenesis (Dong et al. 2025). It directly modulates purine absorption and urease synthesis, regulating UA metabolism (Zhao et al. 2022). Targeting microbiota with SCFA‐producing or purine‐degrading lactic acid bacteria thus represents a therapeutic strategy for inflammatory/allergic diseases (Baquerizo Nole et al. 2014; Furci et al. 2023). Clinical trials have demonstrated that specific strains of lactic acid bacteria, such as *L. gasseri* , *L. salivarius* , *L. johnsonii* , *L. paracasei* , and *L. reuteri* , either individually or in combination with *Bifidobacteria* strains (e.g., *B. animalis* and *B. longum* ), can reduce urticaria severity and improve patients' quality of life (Bi et al. 2021; Dabaghzadeh et al. 2023; Fu et al. 2023). *Lactobacillus* exerts its beneficial effects by inhibiting pathogenic bacteria, regulating immune function, and maintaining immune homeostasis (Yang et al. 2023; Zhang et al. 2023; Kanmani et al. 2013; van Zyl et al. 2020). Notably, certain *Lactobacillus* strains are directly involved in purine metabolism. For instance, *L. gasseri* PA‐3 has been shown to absorb and degrade purine metabolites such as hypoxanthine, inosine monophosphate, and guanosine monophosphate, thereby reducing serum UA levels (Yamada et al. 2017). Similarly, *L. aviarius* CML180 has demonstrated the ability to degrade purine nucleosides, which are precursors for UA production, effectively lowering UA levels in a mouse model of hyperuricemia (Li, Zhang, et al. 2023). While these studies validate the principle that bacterial purine metabolism can influence host UA levels, they focus on metabolic diseases. To explore whether a strain with similar enzymatic activity could benefit an immune‐mediated condition like CSU, we selected *L. paragasseri* LG‐1 for its demonstrated ability to degrade hypoxanthine and UA in vitro. By demonstrating that LG‐1 alleviates urticaria symptoms in a CSU‐relevant model, we extend the application of purine‐metabolising probiotics from metabolic disorders to a complex, immune‐mediated skin disease. Crucially, our whole‐genome sequencing and subsequent analysis not only confirmed the absence of detectable antibiotic resistance or virulence genes but, more importantly, functionally annotated the key genes underpinning its purine‐metabolising capability. This directly links the genomic background of LG‐1 to its core therapeutic mechanism against urticaria. To further validate its efficacy and elucidate its mechanisms of action, an OVA/aluminium hydroxide‐induced urticaria‐like mouse model was established.

As anticipated, in the CSU mouse model, we observed elevated serum UA levels and disrupted gut microbiota. UA is a damage‐associated molecular pattern, which activates innate immune responses by engaging various cell types, including macrophages, monocytes, dendritic cells, MCs, and eosinophils. This activation induces the secretion of pro‐inflammatory cytokines and triggers the NLRP3 inflammasome, initiating an inflammatory response cascade (Joosten et al. 2020; Shin et al. 2020; Desai et al. 2017). UA also functions as an antigen, promoting Th0 cell differentiation into Th2 cells, amplifying allergic inflammation (Hara et al. 2014; Schuler et al. 2020). Since CSU is an IgE‐mediated, MC‐driven type II inflammatory response, elevated UA levels may worsen CSU symptoms. Notably, UA levels dropped significantly following LG‐1 treatment, potentially via gut microbiota modulation and XOR activity inhibition.

Subsequently, urticaria mice exhibited altered β‐diversity and gut microbiota composition. While loratadine treatment reduced α‐diversity, the LG‐1 strain effectively enhanced it. Notably, LG‐1 intervention increased the relative abundance of beneficial bacteria, such as *Lactobacillus* and the Lachnospiraceae family, while reducing the abundance of pathogenic bacteria, including *Enterococcus* and *Pseudomonas*. Furthermore, we observed a negative correlation between UA and hypoxanthine levels and the abundance of *Lactobacillus* and *Bifidobacterium*. The increased presence of harmful bacteria, such as *Enterococcus* and *Pseudomonas*, in the gut may promote LPS production (Wu et al. 2024; Liu, Sun, et al. 2020), which in turn elevates XOR activity and UA levels. Previous studies have demonstrated that SCFAs inhibit XOR and directly degrade UA to acetate, reducing UA production (Li, Li, et al. 2023; Ni et al. 2021; Liu et al. 2023). In our study, we observed elevated XOR activity in both gut and serum samples from urticaria mice; however, this activity was significantly reduced following LG‐1 intervention, consistent with expectations. Additionally, SCFA levels also increased post‐LG‐1 treatment. This suppression of XOR and the concurrent rise in SCFAs are indicative of a microbiota‐mediated indirect mechanism, whereby LG‐1 remodels the gut community to favour bacteria that produce metabolites capable of systemically regulating purine metabolic enzymes. Based on these findings, we hypothesise that a reduction in probiotics responsible for UA degradation and SCFA production may lead to elevated XOR activity, contributing to UA accumulation. Conversely, supplementation with *L. paragasseri* LG‐1, which exhibits hypoxanthine and UA degradation capabilities, represents a promising therapeutic strategy to alleviate CSU symptoms through the gut‐skin axis.

XOR activity is regulated by inflammatory cytokines including IFN‐γ, TNF‐α, and IL‐1 (Li et al. 2022; Wang et al. 2022). Urticaria pathogenesis is driven by type II inflammation, associated with NF‐κB pathway cytokines TNF‐α and IL‐1β (Hu et al. 2023; Sun et al. 2023). NF‐κB, key in inflammatory skin diseases, activates when dimers translocate to the nucleus upon stimulation, driving inflammatory cytokine production (Su et al. 2023). The NF‐κB signalling pathway is primarily activated by TNF‐α, IL‐1β, LPS, and antigens, which bind to cell surface receptors and initiate signalling through various bridging proteins. Additionally, Toll‐like receptor (TLR) family members recognise microbial antigens and further contribute to NF‐κB activation (Guo et al. 2024). Concurrently, the accumulation of UA induces excessive ROS production, which triggers inflammatory processes by promoting the synthesis and secretion of pro‐inflammatory cytokines (Kimura et al. 2021; Hussain et al. 2016). In urticaria mice, elevated IL‐4, IL‐10, IL‐1β, TNF‐α, TLR4, and NF‐κB mRNA occurred, with increased TLR4 and NF‐κB protein by IHC. LG‐1 attenuated these markers. Based on these observations, we propose that LG‐1 exerted its therapeutic effects through a multifaceted mechanism that includes reducing pro‐inflammatory cytokine production, suppressing XOR activity, and decreasing UA levels. The attenuation of the TLR4/NF‐κB pathway is a key component of this anti‐inflammatory response. However, given that uric acid is a known potent endogenous activator of this pathway, its inhibition is likely, at least in part, a consequential effect of the primary reduction in UA levels. This collective action mitigates ROS generation and inflammatory damage, thereby contributing to urticaria symptom relief (Figure S4).

In conclusion, *L. paragasseri* LG‐1 supplementation improved urticaria symptoms, likely through a synergistic mechanism involving direct purine degradation and indirect microbiota modulation. This led to reduced systemic UA levels, oxidative stress, and downstream inflammatory signalling, thereby alleviating inflammation. These findings indicate LG‐1 is a novel therapeutic candidate for CSU. Limitations include an unelucidated direct UA‐CSU relationship, requiring further study. Nonetheless, our results provide insights for future CSU research and therapy.

## Author Contributions

**Qiong Wang:** investigation, methodology, validation, software, data curation, writing – original draft. **Zhiming Hu:** investigation, methodology, validation, software, data curation, writing – original draft. **Yuqi Wang:** methodology, formal analysis. **ShuPing Guo:** supervision, visualization. **Xinglian Zhang:** methodology. **Yunqing Ren:** investigation. **Jinjun Li:** visualization, formal analysis. **Xiaoqiong Li:** writing – review and editing, funding acquisition, supervision, project administration. **Hongzhou Cui:** writing – review and editing, funding acquisition, project administration, resources, supervision.

## Funding

This work was supported by Fundamental Research Program of Shanxi Province, 202303021221224; Bethune Charitable Foundation, J202301E036; The Youth Science and Technology Innovation Talent Project Four Batches of Shanxi Province, 2023RC009. The Key Research and Development Plan of Zhejiang Province, 2025C02080; The State Key Laboratory for Managing Biotic and Chemical Threats to the Quality and Safety of Agro‐products, 2021DG700024–ZZ202210.

## Conflicts of Interest

The authors declare no conflicts of interest.

## Supporting information


**Data S1:** mbt270316‐sup‐0001‐Supinfo.zip.

## Data Availability

The 16s datasets presented in this study can be found in the BioProject PRJNA1101664, PRJNA1096288 in the NCBI GenBank database. The whole genome sequencing raw data have been deposited in the NCBI Sequence Read Archive (SRA) under BioProject accession PRJNA1359023. Other data from this study are available within the article and its Supporting Information or from the corresponding author upon reasonable request.
