# Supplementary material for: Lactobacillus paragasseri LG‐1 Alleviates Urticaria‐Like Symptoms in Mice via Modulation of Gut Microbiota, Hypoxanthine and Uric Acid
Source: Microb Biotechnol. 2026 Feb 17;19(2):e70316. doi: 10.1111/1751-7915.70316 (PMC12914086; doi:10.1111/1751-7915.70316)
Supplement: Supplementary file 1 — Data S1: mbt270316‐sup‐0001‐Supinfo.zip. [file MBT2-19-e70316-s001.zip › mbt270316-sup-0001-Supinfo.docx]

**Supplementary Information**

**Supplementary Table 1 Demographic and clinical characteristics of participants.**

| **Features** | **CSU patients（n=35）** | **HCs（n=21）** | ***p*-value** |
| --- | --- | --- | --- |
| Age (years), mean ± SD | 37.5±13.9 | 39.9±9.4 | 0.40 |
| Sex（famale）*,n（%）* | 20(57.1) | 13（61.9） | 0.79 |
| BMI (kg/m2), mean ± SD | 21.36±1.7 | 20.8±1.6 | 0.10 |
| Time since diagnosis of CSU (months), median (IQR) | 21(3-24) | — | — |
| Age of onset CSU (years), mean ± SD | 34.6±15.3 | — | — |
| Resistance to antihistamines*,n（%）* | 4(11.4) | — | — |
| Gastrointestinal symptoms during the onset of urticaria*,n（%）* | 6(17.1) |  | — |
| History of food allergy*,n（%）* | 10(28.6) | — | — |
| History of drug allergy*,n（%）* | 4(11.4) | — | — |
| Family history of urticaria*,n（%）* | 5(14.3) | — | — |
| Education level*,n（%）* |  |  |  |
| Elementary school | 1(2.8) | — | — |
| Middle school | 5(17.1) | — | — |
| High school | 10(28.6) | — | — |
| College and above | 19(51.4) | — |  |
| Eating habits*,n（%）* |  |  |  |
| Low-fat and high-carbohydrate diet | 19(54.3) | — | — |
| Medium fat and medium carbohydrate diet | 15(42.9) | ­­— | — |
| High-fat and low-carbohydrate diet | 1(2.8) | — | — |
| Place of permanent residence*,n（%）* |  |  |  |
| Rural areas | 5(14.3) | — | — |
| City | 30(85.7) | — | — |
| Helicobacter pylori positive*,n（%）* | 13(37.1) | 0 | 0.002 |
| Keep pets at home*,n（%）* | 4(11.4) | — | — |

CSU, chronic spontaneous urticaria; HCs, healthy controls; BMI, body mass index; IQR, interquartile range.

**Supplementary Table 2 List of primers for markers in mouse skin tissue**

| **Name** | **Primer** | **Sequence (5´-3´)** |
| --- | --- | --- |
| GAPDH | Forward primers | TGCACCACCAACTGCTTAGC |
|  | Reverse primers | GCATGGACTGTGGTCATGAG |
| IL-4 | Forward primers | TACCAGGAGCCATATCCACGGATG |
|  | Reverse primers | TGTGGTGTTCTTCGTTGCTGTGAG |
| IL-10 | Forward primers | GCCCTTTGCTATGGTGTC |
|  | Reverse primers | TCTCCCTGGTTTCTCTTCC |
| IL-1β | Forward primers | GGATATCCTTTCACTCTGCATGGT |
|  | Reverse primers | TGGAGTAAATGGCTATCTCCAGGTA |
| TNF-α | Forward primers | GAAACACAAGATGCTGGGA |
|  | Reverse primers | TTGCAGAACTCAGGAATGG |
| NF-κB | Forward primers | ATGGCAGACGATGATCCCTAC |
|  | Reverse primers | CGGAATCGAAATCCCCTCTGTT |
| TLR4 | Forward primers | ATGGCATGGCTTACACCACC |
|  | Reverse primers | GAGGCCAATTTTGTCTCCACA |


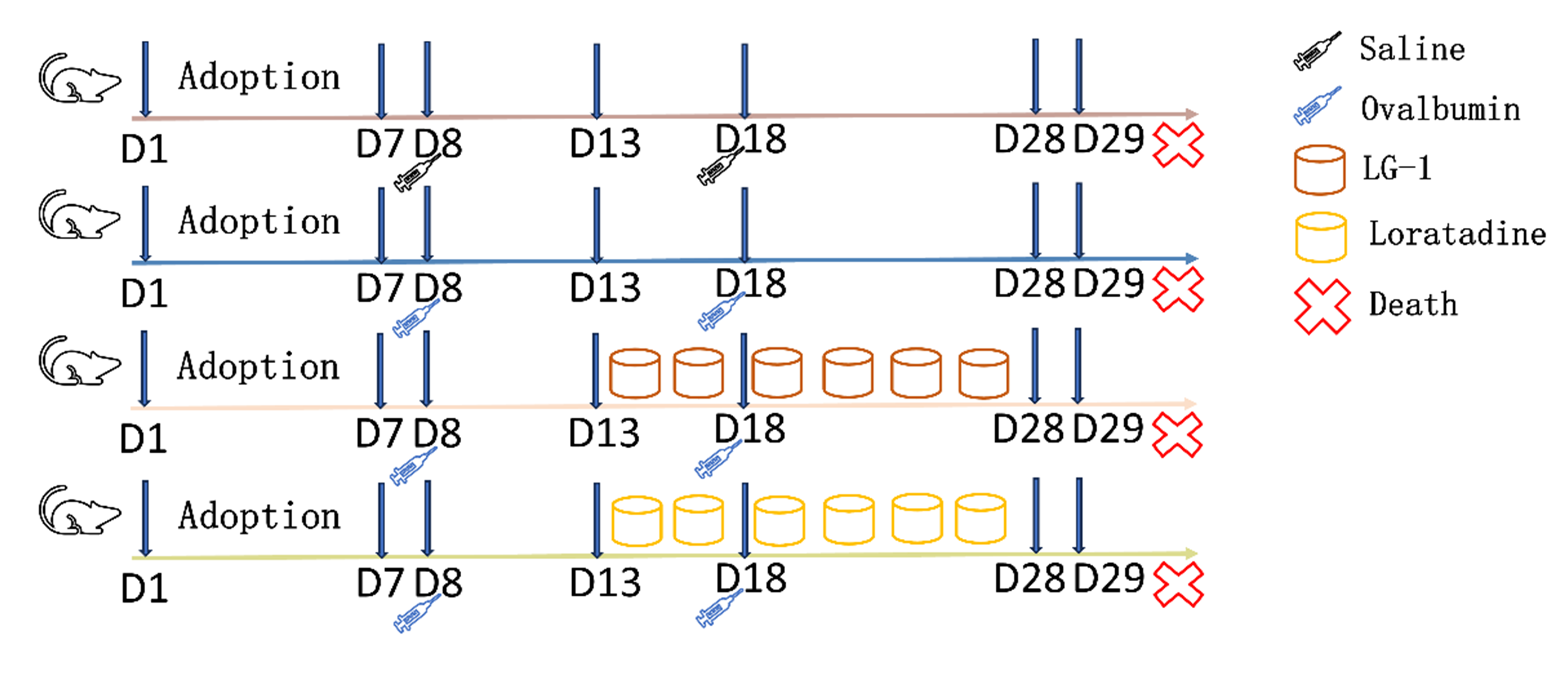


**Supplementary Fig.1 Experimental design for the mouse model**

**
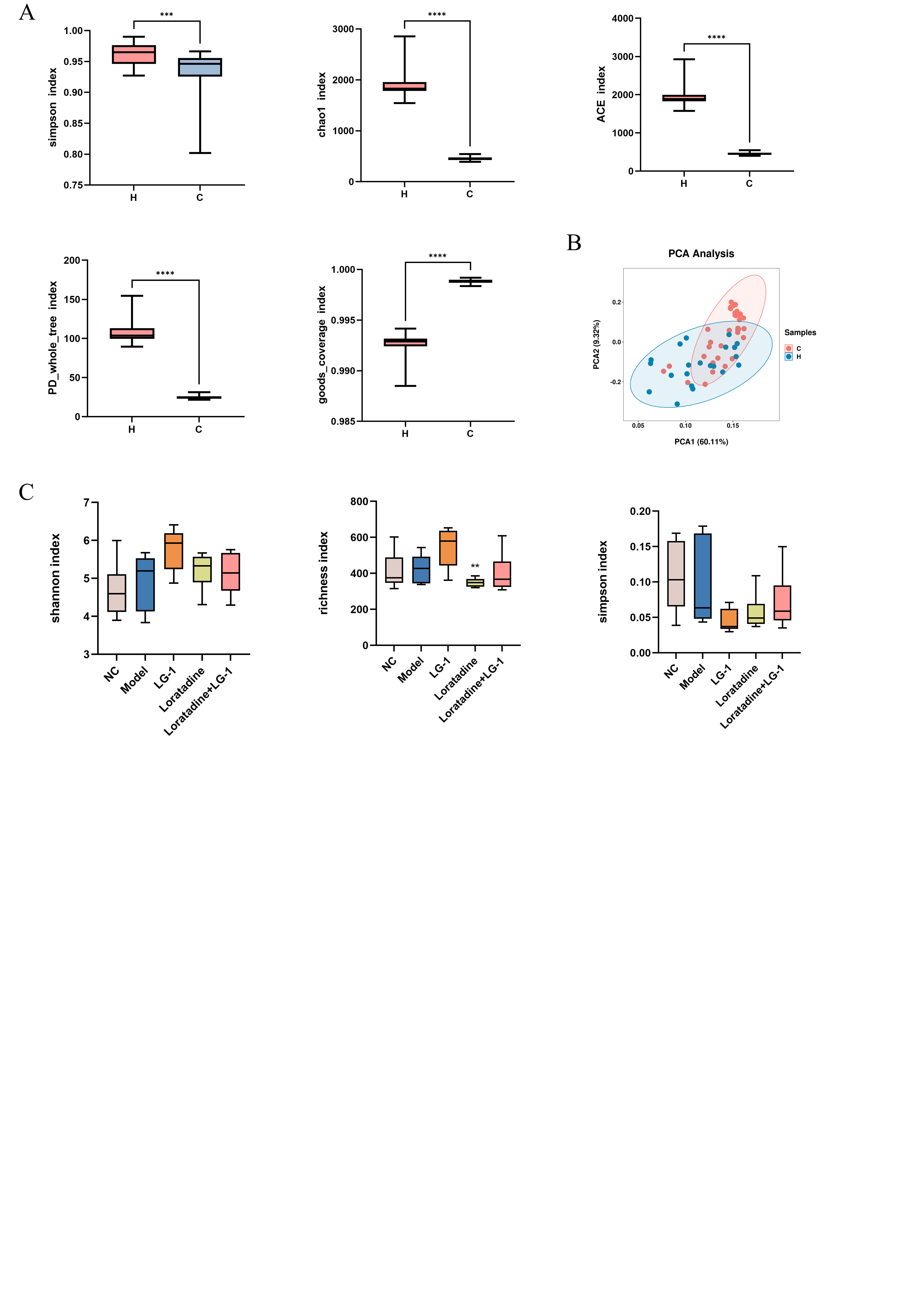
**

**Supplementary Fig.2 Comparing the diversity of the gut microbiota of patients with chronic spontaneous urticaria (C) and healthy controls (H). (A)** α-diversity of gut microbiota. **(B)** Principal Co-ordinates analysis (PCoA) on gut microbiota structures. *** *p* < 0.001, **** *p* <0.0001.

**
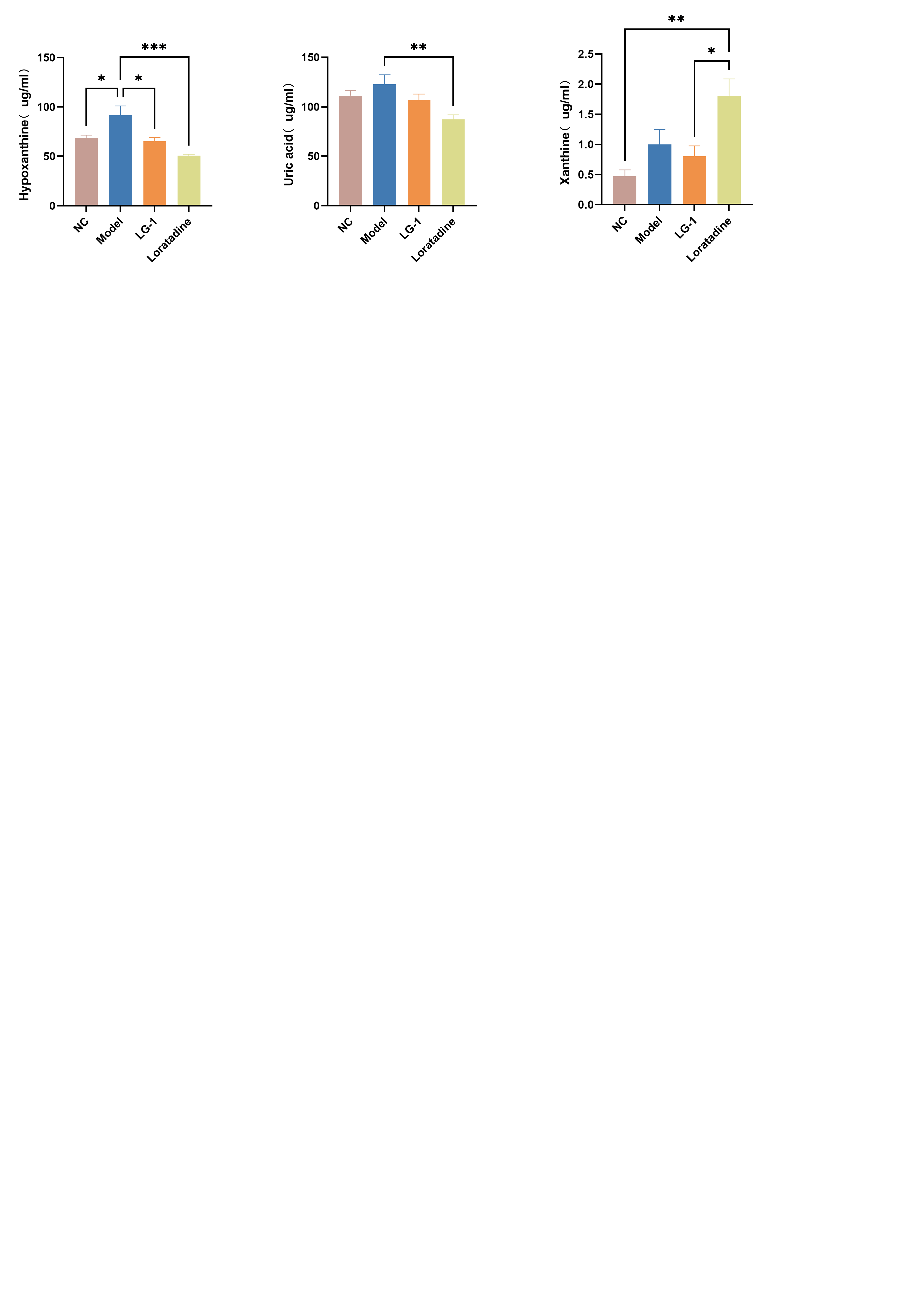
**

**Supplementary Fig.3 The content of purine metabolites uric acid, hypoxanthine, xanthine in mouse feces.** * *p* < 0.05, ** *p* < 0.01, *** *p* < 0.001.


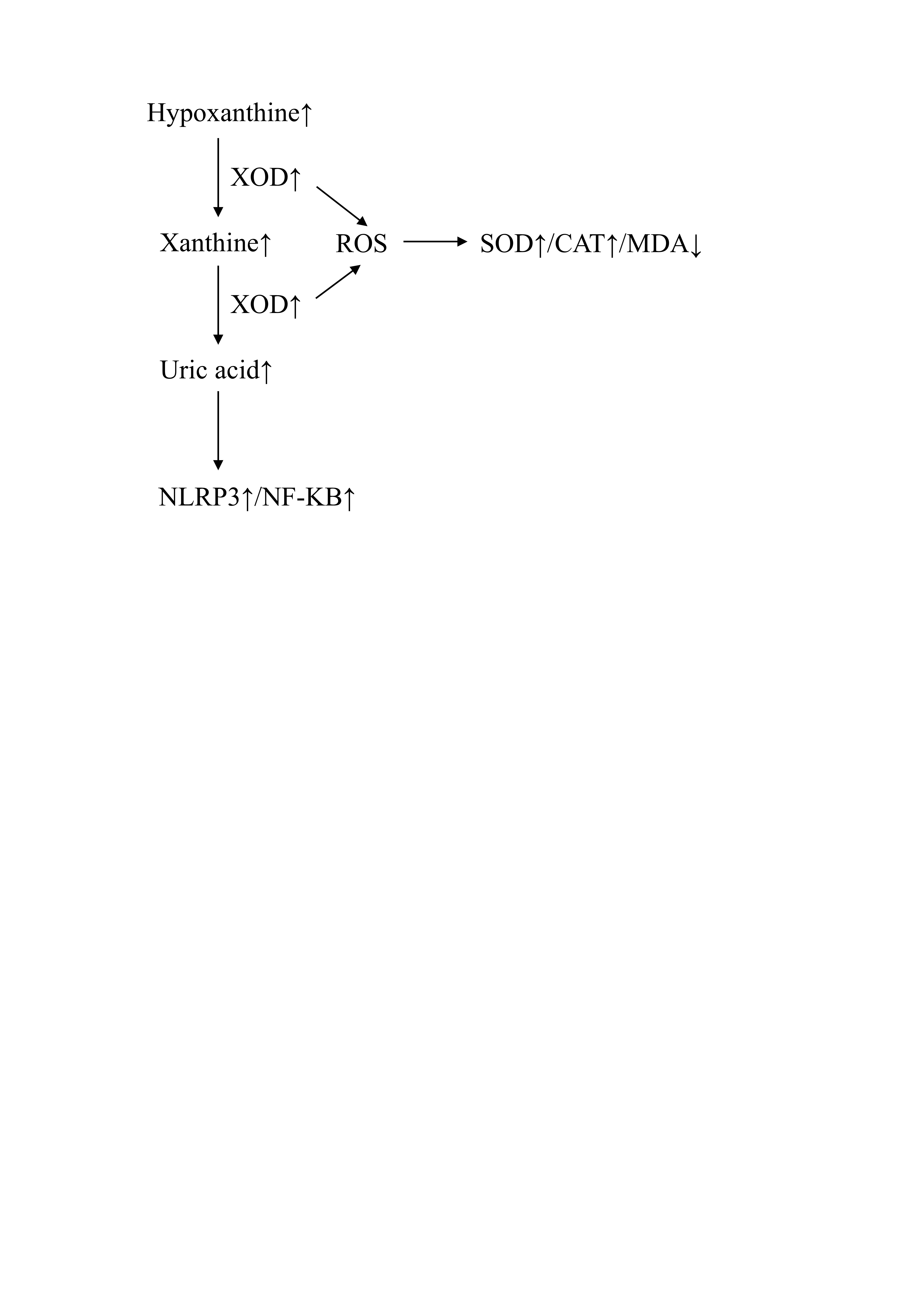


**Supplementary Fig.4 The mutual influence of purine metabolism and oxidative stress.**
